# The diagnostic accuracy of quantitative CMR perfusion imaging may not be the same for all coronary arteries

**DOI:** 10.1186/1532-429X-14-S1-P8

**Published:** 2012-02-01

**Authors:** Geraint Morton, Amedeo Chiribiri, Masaki Ishida, Andreas Schuster, Shazia T Hussain, Eike Nagel

**Affiliations:** 1 King's College London, London, UK

## Background

Absolute quantification of myocardial perfusion with cardiac magnetic resonance (CMR) is an emerging technique and its diagnostic accuracy remains to be fully established. Furthermore it is not known whether there are any differences in the accuracy of coronary artery disease (CAD) detection between coronary arteries.

## Methods

41 patients with known or suspected CAD underwent 1.5T perfusion imaging prior to coronary angiography.

### Perfusion imaging

k-t balanced Turbo Field Echo sequence, shortest TE (range 1.35-1.54ms), shortest TR (range 2.64-3.12ms), 50°flip angle; 90° prepulse, 100ms prepulse delay and typical acquired resolution 1.7x1.9x10mm. 3 short axis slices were acquired every heartbeat. Stress imaging preceded rest by 14±2 minutes. A dual bolus (0.01mmol/kg then 0.1mmol/kg 20 seconds later) of contrast agent was used.

Blinded experts performed all data analysis. Mean segmental perfusion values were obtained using dedicated prototype Philips ViewForum software and Fermi deconvolution. Each segment was assigned to a perfusion territory according to standard definitions. Myocardial perfusion reserve (MPR) was defined as stress perfusion divided by rest perfusion. The mean of the 2 lowest MPR for each perfusion territory was used for analysis.

### X-ray angiography

Quantitative coronary angiography (QCA) was performed on all arteries ≥2mm and a mean diameter stenosis ≥70% regarded as significant. The accuracy of MPR to detect significant CAD as defined by QCA was determined by receiver-operating characteristic (ROC) curves.

## Results

Mean age was 63±9. 32 (78%) participants were male, 13 (32%) diabetic and 30 (73%) hypertensive. 5 (12%) had previous myocardial infarction. LVEF was 63±12%. 25 patients had significant CAD involving 7 left anterior descending (LAD) arteries, 12 circumflex arteries (3 dominant) and 13 right coronary arteries (RCA; 12 dominant).

Rest perfusion was 1.03±0.3ml/min/g in stenotic and 1.06±0.33ml/min/g in remote territories. Corresponding stress perfusion values were 1.54±0.34ml/min/g and 1.94±0.59ml/min/g and MPR 1.31±0.3 and 1.7±0.42.

**Figure 1:**
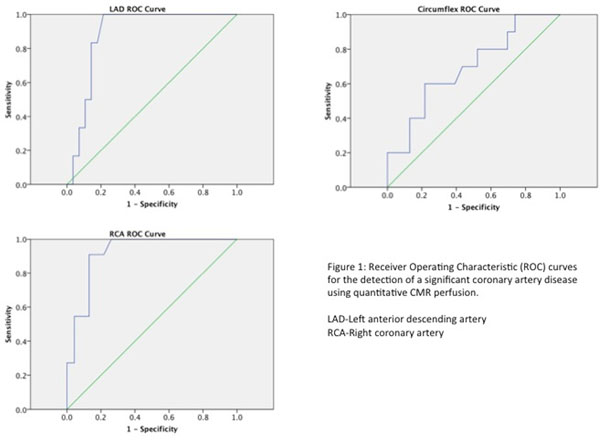


Area under the ROC curve for MPR to detect significant CAD was 0.83 (0.74-0.92). MPR ≤1.45 detected CAD with 82% sensitivity and 81% specificity. ROC curves for each individual coronary artery are shown in figure 1.

Area under the ROC curve, sensitivity and specificity were highest for the RCA and lowest for the circumflex (table 1). Area under the ROC curve was significantly lower for the circumflex artery compared with the RCA (p=0.04) and non-significantly lower (p=0.1) compared with the LAD. There was no significant difference between the LAD and RCA ROC curves (p=0.6).

**Table 1:** 

	Area under ROC curve	95% confidence interval	Optimal MPR cut-off	Sensitivity	Specificity
**Overall**	0.83	0.74-0.92	≤1.45	82%	81%
**LAD**	0.88	0.77-1.0	≤1.35	83%	86%
**Circumflex**	0.69	0.5-0.89	≤1.45	60%	78%
**RCA**	0.92	0.8-1.0	≤1.45	91%	87%

LAD: left anterior descending artery RCA: right coronary artery MPR: myocardial perfusion reserve.

## Conclusions

Quantitative CMR perfusion imaging is accurate for the detection of significant CAD however accuracy is significantly reduced in the circumflex artery. This may be due to reduced signal from the lateral wall or due to incorrect allocation of segments to perfusion territories.

## Funding

This work was supported by a European Union Grant (Grant number 224495 to GM, EN); the British Heart Foundation (Research Excellence Award RE/08/003 and FS/10/029/28253 to AS, EN); the Biomedical Research Centre (grant number BRC-CTF 196 to AS, SH, EN) and the Wellcome Trust and EPSRC (grant number WT 088641/Z/09/Z to AC, EN).

